# Sub-clinical middle ear malfunctions in elderly patients; prevalence, pattern and predictors

**DOI:** 10.4314/ahs.v17i4.34

**Published:** 2017-12

**Authors:** Olusola Ayodele Sogebi, Taiwo Olugbemiga Adedeji, Olatundun Ogunbanwo, Emmanuel Abayomi Oyewole

**Affiliations:** 1 Department of Surgery, OACHS, Olabisi Onabanjo University, Sagamu, Nigeria; 2 Department of Otorhinolaryngology, Head and Neck Surgery LAUTECH Teaching Hospital, Osogbo, Nigeria; 3 Department of ENT, Olabisi Onabanjo University Teaching Hospital, Sagamu, Nigeria

**Keywords:** Middle ear malfunctions, elderly patients

## Abstract

**Background:**

Little is known about functioning of the middle ear with advancing age.

**Objectives:**

To estimate the prevalence and describe tympanometric patterns of sub-clinical middle ear malfunctions,( S-MEM) in elderly patients. It also assessed clinical factors that could predict S-MEM.

**Methods:**

Cross-sectional, analytical study of patients aged ≥ 60 years in a tertiary hospital in Nigeria between 2011–2014. Pure tone audiometry (PTA), tympanometry and acoustic reflexes were recorded. S-MEM was based on audiometric and tympanometric evident abnormalities. Descriptive, univariate and multivariate analyses performed to detect independent clinical predictors of S-MEM at p-value of <0.05.

**Results:**

121 patients , M: F of 1.1:1. Mean age was 70.1 ± 6.2 years, 77.7% were married. Prevalence of S-MEM was 21.5%. Abnormal tympanometric tracings were type A_S_>C>B>A_D_. The parameters that were statistically-significant on univariate analyses were subjected to logistic regression analysis which confirmed previous head injury, diabetes, osteoarthritis of knee joint, and absent acoustic reflex as clinical predictors for S-MEM.

**Conclusion:**

21.5% of elderly Africans had subclinical abnormalities in their middle ear functioning, mostly with type AS tympanogram. Independent clinical predictors of S-MEM included previous head injury, diabetes, history of osteoarthritis of knee joints, and absent acoustic reflex.

## Introduction

Hearing impairment has been reported to be one of the common chronic medical conditions which can reduce quality of life among the elderly. Hearing impairment is grossly classified as conductive, sensorineural or mixed in type. Substantial emphasis has been laid, with concomitant research performed regarding age related-changes presenting with sensorineural hearing impairment^1^–^3^. Thus information and documentation on age-related hearing impairment (ARHI), and factors associated with it are substantial in the medical literature^3^–^5^. Except for common diseases like otitis media of different types, perhaps little is known about the functioning of the middle ear among elderly subjects^6^. Middle ear structures are mainly concerned with transmission of sound waves from the external auditory canal, through the tympanic cavity to the inner ear. This function is performed by three bony ossicles which articulate with each other, one of which is attached to the tympanic membrane (stimulated from the external ear), and another one to the oval window (transmits to the inner ear). Furthermore, the middle ear structures have protective mechanism of binaural acoustic reflexes that prevent unduly loud and hazardous sounds from getting transmitted fully into the inner ear.

The most commonly used method for evaluating middle ear function is by impedance audiometry (tympanometry) 7, which depicts specific types of graphs in different middle ear diseases. The tympanometry tracings, as classified by Jerger can have either of five distinct patterns: A (Normal), A_S_, A_D_, B and C^8^. Except for the A pattern, all the other patterns should manifest with air-bone gaps evidence of conductive hearing impairment on pure tone audiometry. It was however observed on routine clinical evaluation of our elderly patients that there were some of them who although had no clinically evident conductive hearing loss, had audiometric changes suggestive of such. We regarded such patients as having subclinical middle ear malfunctions (S-MEM). S-MEM will aggravate sensorineural hearing impairment which is common among the elderly, thus making rehabilitation of hearing more difficult and further reducing quality of life among elderly subjects.

This study aimed to estimate the prevalence and describe the tympanometric patterns of S-MEM in elderly patients. It also assessed clinical factors that could independently predict S-MEM in the elderly.

## Methods

**Study design:** This was a cross-sectional, analytical study that was conducted among elderly patients attending the Ear Nose and Throat (ENT) clinics of Olabisi Onabanjo University Teaching Hospital, Sagamu Nigeria between January 2011 and December 2014. Consecutive elderly patients aged sixty years and above who attended the clinics were approached as participants in the study. The general nature, significance, requirements of the patients as well as the fact that a decline to participate in the study would not affect treatment was emphasized to the patients.

**Inclusion/exclusion criteria:** Consenting patients were included in the study. Excluded from the study were elderly patients with evidence of inactive or active middle ear diseases on otoscopy like tympanic membrane perforations, current or recurrent discharges from the middle ear, asymmetric audiograms, and incomplete investigations (both forms of audiometry- PTA and tympanometry).

Ethics approval for this study was obtained from the Health Research and Ethics Committee-HREC, of Olabisi Onabanjo University Teaching Hospital, OOUTH.

**Data collection:** Data was collected with intervieweradministered questionnaires divided into three sections. Section A was on questions concerning the socio-demographics of the patients. Section B contained questions relating to the medical conditions of the patients. Otoscopic examination was done in each ear to ascertain the state of the external auditory canal and the tympanic membrane. Tuning fork tests (Rinne and Weber) were done on each of the patients, to confirm clinical evidence of conductive hearing loss in each of the ears.

Section C recorded audiometric profiles of the patients, which included PTA, tympanometry and acoustic reflexes. The acoustic reflex thresholds were tested with contralateral stapedius reflexes for frequencies of 500, 1000, 2000, 4000 Hz; and considered to be normal when the thresholds were between 75 and 110 dB HL.

Normal hearing was regarded as pure tone average (PTAv) of < 25dB HL of both the air and bone conduction hearing thresholds at the measured frequencies (250–4000Hz), with average air-bone gap <10dB HL. Conductive hearing loss was taken as bone conduction PTAv <25, air conduction >25 dB HL with average airbone gap >10dB HL. Mixed hearing loss was taken as PTAv of >25dB HL for both air and bone conduction and also an average air-bone gap >10dB HL. The tympanograms were classified according to Jerger types A, A_S_, A_D_, B and C^8^. Tympanometric findings were divided as (Normal-Jerger's type A tympanogram or Abnormal-other tympanogram types). Presence or absence of acoustic reflexes was also recorded.

Patients were categorized based on the functional status of the middle ear into two groups as either Normal or Abnormal (silent middle ear malfunctioning, S-MEM). S-MEM was regarded as abnormal tympanometric findings with normal tympanic membrane.

**Data presentation and analysis:** Descriptive analysis of the samples was done using frequency tables and graphical representations, while cross-tabulation was prepared to demonstrate the relationship between variables. On univariate analysis, associations between discrete variables were treated using Chi-square test while continuous variables were assessed by the Student's t-test. The clinical parameters that were found to have significant associations on univariate analysis were evaluated as independent clinical predictors of S-MEM by a series of bivariate logistic regression models in which each clinical parameter was treated as an independent predictor and S-MEM was treated as outcome variable. The level of statistical significance was set at p-value of <0.05 for all analyses. The data analysis was done using the SPSS version 19.0 (Chicago, Il)

## Results

One hundred and twenty one patients participated in this study which comprised of 52.1% males, M: F = 1.1:1. The age ranged from 61 to 96 with a mean of 70.1 ± 6.2 years. The details of the demographic characteristics of the patients is shown in Table 1.

**Table 1:** Demographic characteristics according to Sex of the patients

Characteristic	Male	Female
**Age group (Years)**	n=63(52.1%)	n=58 (47.9%)
61–65	15 (12.4)	15(12.4)
66–70	24 (19.8)	18 (14.9)
71–75	17 (14.6)	12 (9.9)
76–80	4 (3.3)	9 (7.4)
≥ 81 3	(2.5)	4 (3.3)
**Marital status**		
Married	50 (41.3)	44 (36.4)
Others	13 (10.7)	14 (11.6)
**Level of education**		
Less than secondary school	17 (14.0)	13 (10.7)
Secondary school and above	46 (38.0)	45 (37.2)
**Occupational class**		
Unskilled	18 (14.9)	12 (9.9)
Partially skilled	29 (24.0)	23 (19.0)
Skilled/Professional	16 (13.2)	23 (19.0)

**Figure 1:**
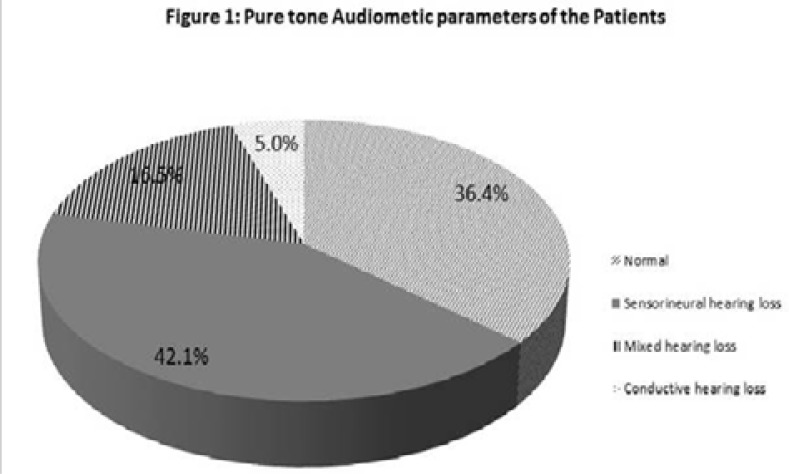
Pure tone Audiometic parameters of the Patients

The middle ear functions assessment by Pure Tone audiometric parameters in Figure 1 revealed S-MEM either as conductive or as mixed hearing loss. Twenty six patients (constituting 21.5%) had S-MEM according to the defined criteria.

**Figure 2:**
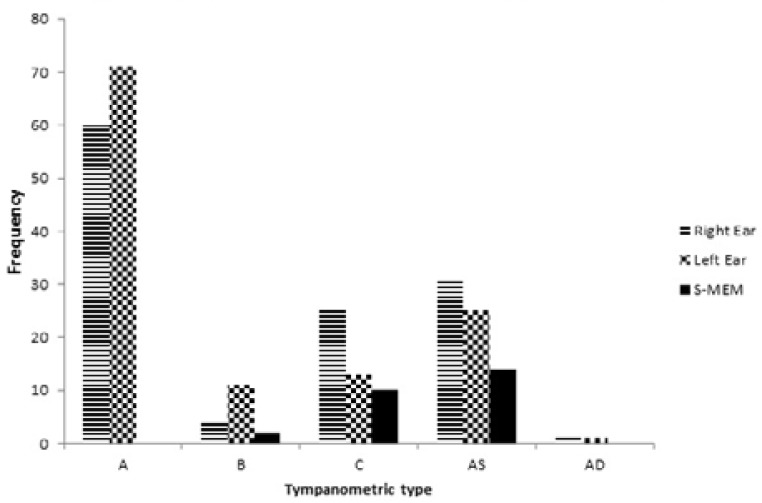
Tympanometric Profile of the Patients (Jerger's classification)

Figure 2 depicts the tympanometric patterns of all the patients according to the ears. The patterns of S-MEM obvious on tympanograms as any other pattern aside from the type A tympanogram.

The patterns of abnormal tympanometric tracings was the type A_S_, followed by types C, B and A_D_ in decreasing frequencies. Table 2 is the comparison of the clinical characteristics of the patients in relation to the functioning of the middle ear. There were no statistically-significant differences in the demographic and some social history parameters, whereas occupational class, previous noise exposure, previous ear discharge, history of previous head injury, diabetes, osteoarthritis of the knee joint, prolonged medications usage, and absent acoustic reflex had statistically-significant differences on univariate analysis.

**Table 2:** Comparison of clinical parameters in relation to middle ear function of patients

Variable	Middle ear function			
	Normal (%)	Abnormal (%)		
	N=95 (78.5%)	N=26 (21.5%)	Statistic	p-value
*Age (Mean±SD)	69.9±5.9	71.2±7.0	1.063	0.290
Sex (Male)	40.5	11.6	0.042	0.838
Marital status (Others)	19.0	3.3	0.917	0.338
Education (≥Secondary)	59.5	15.7	0.081	0.777
Occupation (Skilled)	37.3	50.0	4.823	0.030
Family history of hearing loss	12.4	5.0	0.756	0.390
Social history				
Alcohol consumption (Yes)	33.1	8.3	0.112	0.738
Smoking (Yes)	9.9	3.3	0.135	0.746
Noise exposure (Yes)	19.8	12.4	9.828	0.002
Medical history				
Previous ear discharge	11.6	7.4	5.240	0.044
Head injury	5.8	8.3	16.343	<0.001
Hypertension	29.8	6.6	0.448	0.503
Diabetes	11.6	9.1	9.466	0.002
Osteoarthritis	12.4	10.7	13.433	<0.001
Medications Ototoxic drugs	19.8	6.6	0.318	0.573
Prolonged medications	21.5	11.6	6.467	0.011
Acoustic reflex (Absent)	22.3	14.0	12.053	0.001

* Statistic is Student t-test

Further exploration of the significant clinical parameters on univariate analyses, controlling for age and sex, with multivariate logistic regression analysis revealed previous head injury, diabetes, osteoarthritis of the knee joint, and absent acoustic reflex were confirmed as clinical predictors for S-MEM in elderly patients. The other parameters (occupational class, exposure to noise, previous ear discharge and prolonged medications, were not clinical predictors. This is shown in Table 3.

**Table 3:** Logistic regression analysis to ascertain Clinical predictors of silent middle ear malfunction (control for Age and Sex)

Variable	OR (95% C.I.)	P-value
Occupation	1.024(0.153–6.863)	0.980
Noise exposure	2.507 (0.653–9.619)	0.180
Previous ear discharge	3.788 (0.704–20.372)	0.121
Head injury	8.061 (1.380–47.077)	0.020*

Diabetes	4.571 (1.091–19.147)	0.038*
Osteoarthritis	5.737 (1.483–22.196)	0.011*
Prolonged medications	1.980 (0.547–7.168)	0.298
Absent acoustic reflex	5.026 (1.404–17.994)	0.013*

* Statistically-significant

## Discussion

In clinical practice it is generally assumed that when features of middle ear disease are absent, hearing impairment in elderly subjects are usually due to sensorineral hearing loss (SNHL), especially age related hearing loss, (ARHL). It is important that the functioning of the middle ear be assessed and clarified even when the index of suspicion for SNHL is high. The fact that one in every five (21.5%) of our elderly patients had S-MEM gives credence to this.

Detection of S-MEM may not significantly affect the management plan of hearing impaired elderly subjects which include sound amplifications through use of hearing aids, cochlear implants and assisting listening devices^9^,^10^. However its discovery will assist the audiologist or otologist in having a clearer picture, and a better understanding of the patient's problems and needs. Moreover, there are assertions that malfunctions of the middle ear affects inner ear functioning. For instance, middle ear measurements by broadband middle ear power reflectance (BMEPR) suggested middle ear transmission characteristics played a role in active physiologic process within the inner ear, measured by spontaneous otoacoustic emissions (SPOAEs)^11^. Similarly, Uchida et al confirmed that the condition of the middle ear function measured by tympanometry affects the functioning of the cochlear measured by distortion-product otoacoustic emissions (DPOAEs)^12^ It is thus important that middle ear dynamics and functioning be assessed in order to effectively manage other forms of hearing impairment in elderly subjects.

The prevalence rate of 21.5% for S-MEM as found in this study is within the range 20.0–30.0% for ARHL reported among elderly patients of up to 70 years in Europe^13^. This evokes a natural inquiry into ascertaining if S-MEM is age-related or not. Opinions differ on whether there are changes in the functioning of the middle ear as age advances. In a study on human subjects aged 48–92 years, on the prevalence and 10-year incidence of 4-kHz air-bone gaps (an indicator for middle ear malfunction) and associated factors, it was reported that prevalence increased with age, and that a finding of a 4-kHz air-bone gap may reflect a combination of aging and other factors^14^ Wiley et al, assessed tympanometric measures in older adults, and reported that relative to younger adults, tympanometric measures for older adults showed greater variability, and other features of middle ear malfunctioning^15^. Other studies however did not find consistent affectation of the middle ear functions as ageing occurs^16^,^17^. The foregoing only leads us to conclude that there may be age-related changes in the middle ear functioning which might not be fully clarified yet.

The most common pattern of middle ear malfunctioning represented by tympanometric measures was type A_S_ which signifies reduced movement of the middle ear ossicles. This tympanometric type is likely to result from tympanosclerosis with mechanical impairment of the ossicular movements^18^. This could also be a consequence of diseases of the ossicular joints with attendant ankylosis. In the middle ear, cartilage exists within the three ossicular joints^19^ and their inflammation will manifest with reduced movement represented by type A_S_ tympanogram. Type A_s_ tympanogram was also the most common type of tympanometric pattern seen in patients with idiopathic juvenile arthritis^20^. Moreover in patients with rheumatoid arthritis, there was evidence of sensorineural type of hearing loss as well as diminished compliance in the middle ear, which was not at a level of a conducting type hearing loss^21^. These observations resonate with the findings in this study.

C typmpanogram representing eustachian tube dysfunction was the next common pattern of S-MEM in this study. The tensor veli palatini muscle (TVPM) attaches to the eustachian tube (ET) and reasonably controls its functioning. Suzuki et al., found that the length of the TVPM attachment and its ratio to the length of the ET, especially that of its cartilaginous portion, decreases with age from young adulthood to later life. These findings were thought to be related to postnatal development and aging^22^.

The clinical parameters that were associated with abnormal middle ear functioning were varied. Contrary to the report that middle ear mobility of males decreased gradually with an increase in age^6^, we found neither age nor sex to be significantly associated with subclinical middle ear malfunctioning in this study. The acoustic stapedius reflex ASR allows the stapedial muscle in each ear to respond to ipsilateral, contralateral, and binaural stimulation^23^. When there is impaired hearing affecting the speech sounds predominantly, there is impairment of the acoustic reflex. Furthermore, abnormal contraction of the tensor tympani results in a low-tone hearing loss, with a decrease in middle ear compliance^24^. A higher prevalence of S-MEM and hearing loss could be expected in osteoarthritis due to degeneration of the cartilage at the ossicular joints and subsequent abnormal repair response^25^. It should also produce a type AS tympanogram as expatiated earlier in the discourse. Osteoarthritis was predictive of S-MEM in this study.

Similarly, previous head injury was predictive of S-MEM in elderly patients. A head injury especially when associated with loss of consciousness most likely had an associated injury to the brain, even if subtle. The effect on the middle ear may be linked indirectly to the central-mediation of the acoustic reflexes. There is evidence of impaired affective modulation of the startle reflex following traumatic brain injury (TBI)^26^. Moreover, a significant head injury could be associated with fractures of the base of skull^27^ and associated ossicular chain disruption predisposing to abnormal middle ear functioning^28^. Previous bleeding into the middle ear cavity (hemotympanum) can also predispose to tympanosclerosis^29^.

The effects of diabetes on the middle ear which are well known include different forms of otitis media with complications, and extension of necrotizing otitis externa^30^. However, Tuz et al, reported necrosis of incus and stapes suprastructure associated with diabetes^31^. It is difficult to explain the predictability of diabetes for S-MEM for now, and this necessitates further studies on the effects of this multisystemic disease on the ear.

Some limitations were noted in this study and they include the following. A snap shot at the functioning of both the middle and inner ear may not give a complete picture about their functioning and abnormalities, which may change over time. The limitation in reliability of conventional tympanometer in analyzing middle ear function is also acknowledged. Despite these limitations, this study has been able to depict the burden of S-MEM in elderly African patients, discover the patterns and the independent clinical predictors for these abnormalities.

## Conclusion

21.5% of elderly native Africans had sub-clinical abnormalities in their middle ear functioning, mostly with impaired ossicular mobility pattern. The independent clinical predictors of these malfunctions included previous head injury, diabetes, a history of osteoarthritis of the knee joints, and absent acoustic reflex.
